# Optogenetic fMRI for Brain-Wide Circuit Analysis of Sensory Processing

**DOI:** 10.3390/ijms232012268

**Published:** 2022-10-14

**Authors:** Jeong-Yun Lee, Taeyi You, Choong-Wan Woo, Seong-Gi Kim

**Affiliations:** 1 Center for Neuroscience Imaging Research (CNIR), Institute for Basic Science (IBS), Suwon 16419, Korea; 2 Department of Biomedical Engineering, Sungkyunkwan University, Suwon 16419, Korea; 3 Department of Intelligent Precision Healthcare Convergence, Sungkyunkwan University, Suwon 16419, Korea

**Keywords:** optogenetics, functional magnetic resonance imaging (fMRI), optogenetic fMRI (ofMRI), sensory system, sensory processing

## Abstract

Sensory processing is a complex neurological process that receives, integrates, and responds to information from one’s own body and environment, which is closely related to survival as well as neurological disorders. Brain-wide networks of sensory processing are difficult to investigate due to their dynamic regulation by multiple brain circuits. Optogenetics, a neuromodulation technique that uses light-sensitive proteins, can be combined with functional magnetic resonance imaging (ofMRI) to measure whole-brain activity. Since ofMRI has increasingly been used for investigating brain circuits underlying sensory processing for over a decade, we systematically reviewed recent ofMRI studies of sensory circuits and discussed the challenges of optogenetic fMRI in rodents.

## 1. Introduction

The sensory system generally consists of visual, auditory, olfactory, gustatory, tactile, proprioceptive, and vestibular systems in addition to interoception such as pain, itch, temperature, hunger, thirst, and breathing [1]. To understand and respond to the dynamic environment, the brain interprets sensory signal based on locomotion, prior experiences, or emotional states (e.g., arousal and valence) [2]. Therefore, sensory processing goes beyond the sensation of internal or external stimuli and involves cognitive manifestations to produce an appropriate reaction to stimuli. Sensory abnormalities, a common symptom of neurological disorders such as brain injury, developmental and psychiatric disorders, etc., are classified into three patterns [3,4,5]: (i) sensory discrimination disorder with difficulty in perceiving stimuli, (ii) sensory-based motor disorder with difficulty in sensorimotor coordination and motor task, and (iii) sensory modulation disorder with difficulty in responding to stimuli (over/under reaction). Sensory modulation disorders are the most common symptoms of sensory abnormalities, which involve multiple brain regions and are easily affected by other cognitive factors. Thus, it is critical to understand how sensory information is processed at a network level.

Optogenetics is a sophisticated approach for modulating electrical properties of specific neural cells with light by genetically targeting the expression of encoded light-sensitive proteins, which allows for the gain or loss of function in target sites at a precise time window, in live animals [6]. Optogenetics has been applied to animal behavioral experiments and combined with neural recording techniques, such as electrophysiology and calcium imaging, to determine a causal relationship between neural circuits and behavior (see the review article in [7]). Optogenetic functional magnetic resonance imaging (often referred to as ofMRI or opto-fMRI) is a hemodynamic-based imaging method that can indirectly measure neural activity and observe whole-brain circuitry under specific cellular modulation [8,9]. ofMRI provides a unique opportunity for investigating multi-scale neural mechanisms from the local circuit to the network level.

ofMRI was initially reported by Lee et al. (2010) and Desai et al. (2011) in the rodent sensorimotor system [10,11]. Lee et al. (2010) observed BOLD fMRI responses in the stimulated primary motor cortex (M1) and downstream thalamus from optogenetic activation of excitatory cells in anesthetized rats [10]. Desai et al. (2011) showed that BOLD fMRI responses to optogenetic stimulation of excitatory neurons in the primary somatosensory cortex (S1) were similar to that of somatosensory stimulus in awake mice [11]. These early studies demonstrated that ofMRI is a powerful tool to map brain-wide downstream networks responding to excitation at the stimulated site. Since the sensory system was most widely used for ofMRI studies, this review summarizes current knowledge and understanding of ofMRI research related to sensory processing. In addition, potential issues related to ofMRI are discussed.

## 2. Basics of Optogenetics Relevant to fMRI

**Figure 1:**
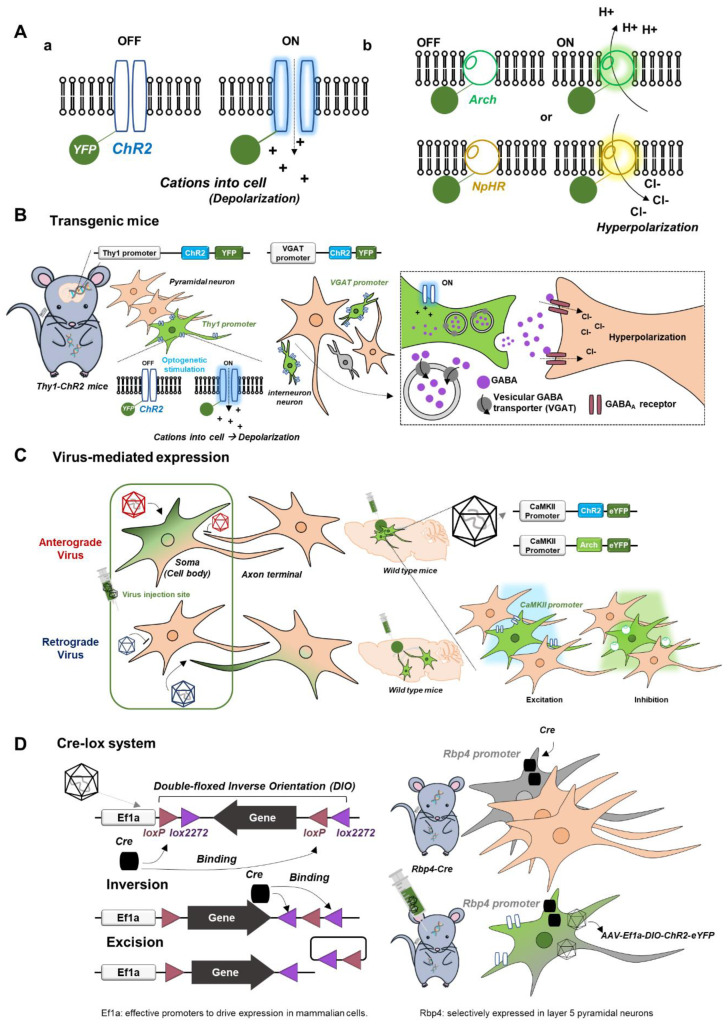
Basics of optogenetics relevant to fMRI. (**A**) Light-sensitive opsins. Channelrhodopsin2 (ChR2; nonspecific cation channel), a representative excitatory opsin, which causes membrane depolarization (**a**). Inhibitory opsins such as halorhodopsin (NpHR; inward chloride pump) and archaerhodopsin (Arch; outward proton pump), which cause membrane hyperpolarization (**b**). (**B**–**D**) Three different approaches for the expression of light-sensitive opsins. (**B**) Transgenic mice with opsins in cell-type specific neurons. Transgenic Thy1-ChR2 mice for optogenetic excitation of pyramidal neurons. Transgenic VGAT-ChR2 mice for optogenetic inhibition of pyramidal neurons. (**C**) Virus-mediated expression of opsins for promotor-based gene delivery into neurons. Anterograde infection from cell bodies to axon terminals. Retrograde infection from axon terminals to cell bodies. (**D**) Cre-lox system for site- and cell-specific optogenetics. Diagram of the double-floxed inverse orientation (DIO) transgenic system.

Since many outstanding review articles already exist [6,7], we briefly describe optogenetic tools relevant to fMRI research (Figure 1). Channelrhodopsin2 (ChR2), a representative excitatory opsin, is a nonspecific cation channel activated by blue light, causing membrane depolarization, while inhibitory opsins such as halorhodopsin (NpHR, Jaws; inward chloride pump) and archaerhodopsin (Arch; outward proton pump) are commonly used for hyperpolarization (Figure 1A). For the expression of light-sensitive opsins, three different approaches are available.

(i). Transgenic mice with opsins in cell-type specific neurons (Figure 1B): for example, transgenic Thy1-ChR2 mice express ChR2 in a specific subpopulation of excitatory pyramidal neurons with the Thy1 promoter, and transgenic VGAT-ChR2 mice express ChR2 in inhibitory interneurons under the vesicular GABA transporter (VGAT) promoter [12,13]. These transgenic mouse lines are engineered to be born with an introduced gene such as ChR2.
(ii). Virus-mediated expression of opsins for promotor-based gene delivery in neurons (Figure 1C): adeno-associated viruses (AAV) or lentiviruses are commonly used. Depending on the type of virus, opsins are transfected anterogradely from soma to axon terminal or retrogradely from axon terminal to soma. For example, calcium/calmodulin-dependent protein kinase II (CaMKII) is a gene expressed only in excitatory pyramidal neurons, so the local injection of AAV-CaMKII-ChR2 (or Arch) in wild-type mice or rats is commonly used for ChR2 (or Arch) expression in excitatory pyramidal neurons.
(iii). Cre-lox system for site- and cell-specific optogenetics (Figure 1D): Cre (recombinase protein) recognizes lox (a unique DNA sequence) to induce site-specific recombination between two lox sites called “floxing,” resulting in inversion, deletion, or translocation. For example, when an AAV vector (e.g., AAV-DIO-ChR2) containing an inverted and double-floxed ChR2 gene is locally delivered into transgenic mice expressing Cre recombinase under a specific promotor, the inverted ChR2 gene will only be flipped to the correct orientation and be functional, resulting in ChR2 expression in a specific cell type with Cre.

## 3. Optogenetic Strategies for fMRI

**Figure 2:**
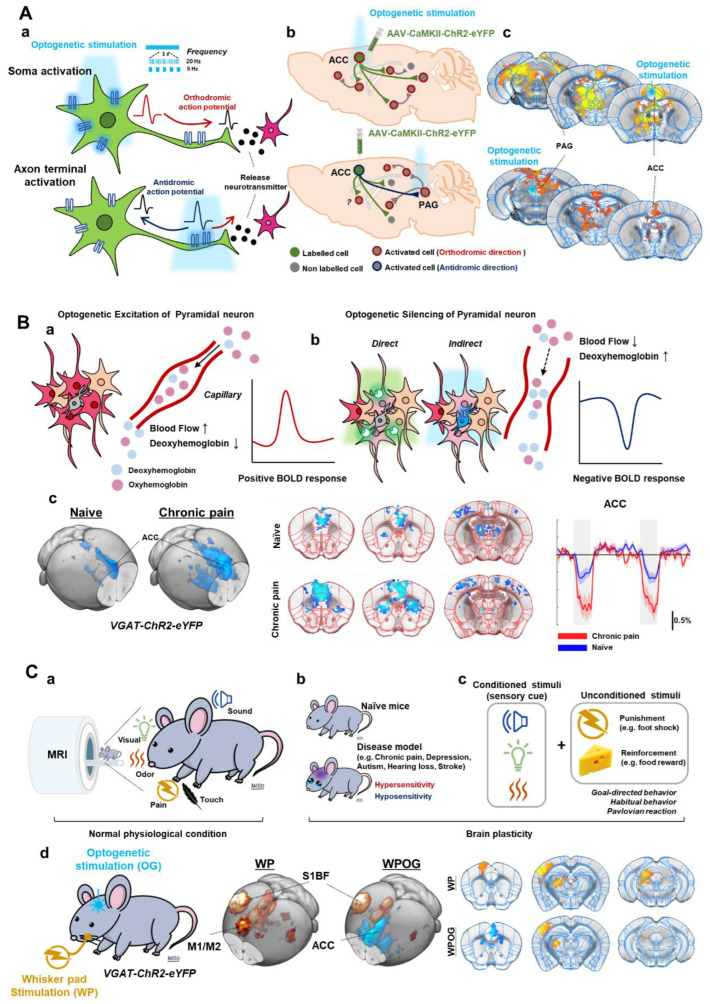
Optogenetic strategies for fMRI. (**A**). Optogenetic stimulation of excitatory cell bodies (soma) or axon terminals. Orthodromic and antidromic propagation responding to the stimulating site (**a**). Postsynaptic activities by optogenetic stimulation (**b**). ofMRI response maps induced by optogenetic stimulation; fMRI response map by cell body stimulation of ACC and by axon terminal stimulation of ACC-PAG projection (**c**). (**B**). Optogenetic silencing by inhibitory opsins in excitatory pyramidal neurons (**a**) or excitatory opsins in inhibitory interneurons (**b**). ofMRI response of naïve and CFA-induced chronic pain model mice during ACC inhibition (**c**). (**C**). Combining optogenetics with sensory-evoked fMRI. fMRI with sensory stimuli (sensory-evoked fMRI) (**a**). Applying sensory-evoked fMRI to animal disease models (**b**) or conditioning behaviors (**c**). Silencing ofMRI combined with electrical stimulation (**d**). Adapted with permission from Lee et al. (2022) [20]. ACC; anterior cingulate cortex, PAG; periaqueductal gray.

Based on previous ofMRI studies [14,15,16,17,18,19,20], three different optogenetic fMRI strategies can be adopted (Figure 2):

(i). Optogenetic excitation of excitatory cell bodies (soma), which is the most common, or axon terminals to map cell-type-specific functional downstream or circuit-specific networks.
(ii). Optogenetic silencing by excitatory opsins in inhibitory interneurons or inhibitory opsins in excitatory pyramidal neurons to measure spontaneous (resting-state) activity in the stimulation site and downstream resting-network strength.
(iii). Modulation of sensory processing by optogenetics. The sensory stimulus can be combined with optogenetic silencing or excitation for dissecting sensory circuits or determining modulatory effects.

### 3.1. Optogenetic Excitation and fMRI: Local Cell Bodies or Axonal Projections

fMRI response to optogenetic excitation allows us to observe the excitatory output of the stimulated region and its downstream pathways at the whole brain level. In the case of soma activation, neural activity propagates in an orthodromic direction along axons (Figure 2(Aa)), followed by a synaptic transmission, which then activates postsynaptic neurons. These polysynaptic responses can be observed by fMRI (Figure 2(Ab)), which has been adopted in most previous ofMRI studies. The optogenetic approach to activating axon terminals can trigger antidromic propagation from axon to soma in addition to postsynaptic activation induced by synaptic transmission (Figure 2A) [21,22]. In a specific example study, AAV-CaMKII-ChR2 was injected into the anterior cingulate cortex (ACC) (Figure 2(Ac)). Soma activation of ACC pyramidal neurons elicited a positive fMRI response in the ACC and its downstream regions including periaqueductal gray (PAG) [20]. Axon terminal activation of ACC-originating pyramidal neurons at PAG also induced a positive fMRI response in the stimulated site PAG and upstream ACC region. In addition, optogenetic activation can mimic various frequency-dependent activities of neurons, which induces heterogeneous brain responses.

### 3.2. Optogenetic Silencing and fMRI: Direct or Indirect Inhibition of Pyramidal Neurons

With the cortex being composed of 80–90% excitatory pyramidal neurons and 10–20% inhibitory interneurons, we can use two strategies for optogenetic silencing of excitatory pyramidal neurons (Figure 2B) [23,24,25,26,27]: (i) expression of inhibitory opsin in excitatory pyramidal neurons or (ii) expression of excitatory opsin in inhibitory interneuron. For example, excitatory pyramidal neurons can be directly suppressed using AAV-CaMKII-Arch. In addition, previous studies showed that the optogenetic stimulation in VGAT-ChR2 mice could robustly increase inhibitory neural activity, which consequently inhibited excitatory pyramidal neurons [17,23,24,25,26]. Thus, both strategies can effectively inhibit excitatory neuronal activities in the stimulated site and excitatory output to downstream pathways [23,24,26].

Although inhibitory neurons can release vasoactive compounds and increase fMRI signals [17], fMRI responses predominantly originate from excitatory neurons. Thus, the optogenetic activation of inhibitory neurons reduces excitatory neuronal activities and, consequently, fMRI signals at both the target site and its downstream regions (Figure 2(Bb)). Thus, ofMRI with neural silencing can determine the strength of interregional communication under basal conditions [17,19,20]. In the chronic pain model, indirect inhibition of the ACC pyramidal neurons (via VGAT-ChR2) enhanced negative BOLD responses in the ACC and its downstream regions (Figure 2(Bc)), suggesting that the chronic pain condition increases spontaneous neural activity in the ACC and enhances interconnected network strengths in downstream regions [20].

### 3.3. Combining Optogenetics with Sensory-Evoked fMRI

fMRI with sensory stimuli (sensory-evoked fMRI) in the rodent is useful for examining brain-wide activity in response to visual, somatosensory, auditory, and olfactory stimuli (Figure 2(Ca)) [14,18,28,29,30]. Sensory-evoked fMRI studies to date have shown results consistent with well-known pathways identified with electrophysiological approaches. For instance, electrical stimulation of the whisker pad induced positive fMRI responses along the major brain regions in the somatosensory pathway (e.g., ventral posterior thalamus (VP), S1, M1, and posterior thalamus (PO)) (Figure 2(Cd)). Applying sensory-evoked fMRI to animal disease models can elucidate the mechanism of hyper- or hyposensitivity (Figure 2(Cb)). For example, in the peripheral nerve injury model, sensory-evoked fMRI response was increased only in S1 but not in the thalamus, which was associated with changes in the synaptic plasticity of thalamic input to S1 [31]. In transgenic mice with neurodevelopmental disorders that lack the neocortical structure, the thalamic fMRI response to somatosensory stimuli was enhanced due to the changes in synaptic transmission of the thalamus [16]. Furthermore, we can also use sensory cues as conditioned stimuli to induce conditioning via reinforcement or punishment (Figure 2(Cc)). Thus, brain-wide fMRI can identify the neural networks involved in emotion, motivation, prediction, and learning [32,33,34].

To further investigate the neurobiological mechanisms and neural circuitry or plasticity, optogenetics can be combined with sensory-evoked fMRI. With ofMRI, we can investigate how a brain region interacts and modulates sensory processing (Figure 2(Cd)). For example, when optogenetic inhibition of the ACC was combined with electrical whisker pad stimulation, we were able to identify that the ACC modulated motor-related areas such as M1 and secondary motor cortex (M2) without affecting sensory areas such as S1 and VP [20]. Thus, sensory-evoked ofMRI is a novel and effective method for investigating the long-range circuit modulation of sensory processing.

## 4. Brain-Wide Optogenetic fMRI in Sensory Processing

**Figure 3:**
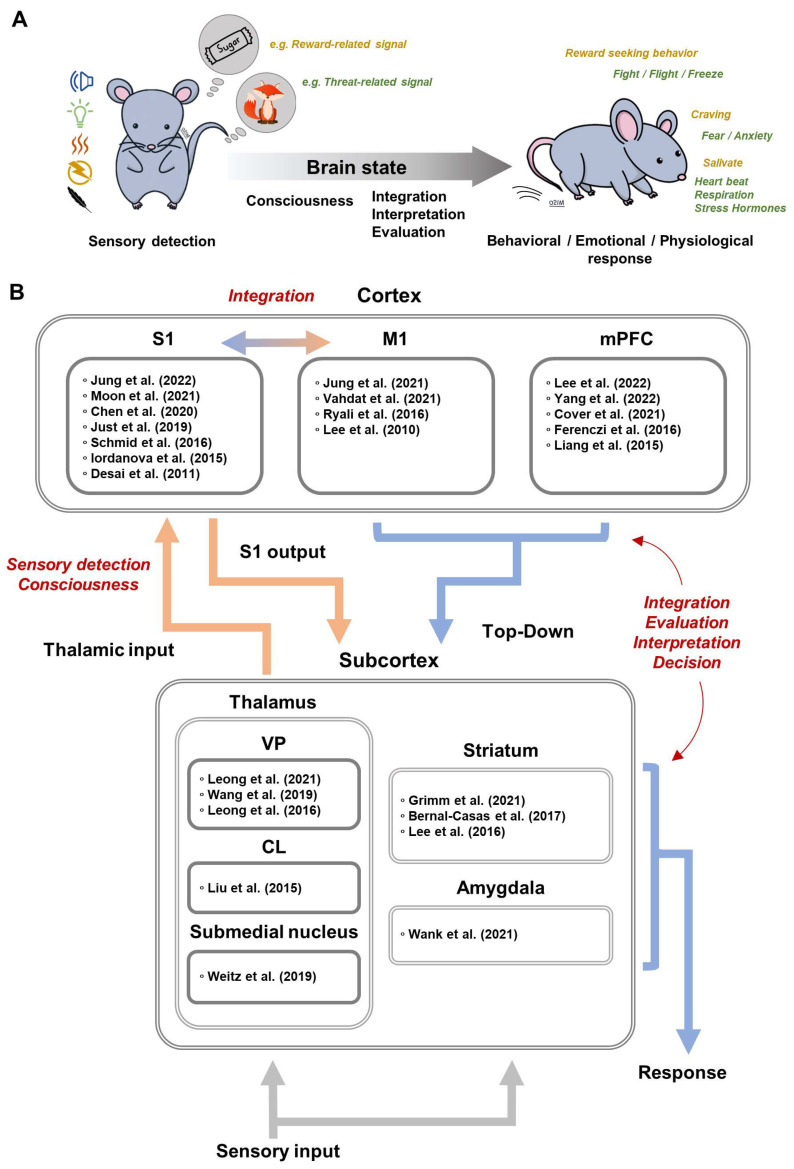
Brain-wide optogenetic fMRI in sensory processing. (**A**). Schematic representation of sensory processing. (**B**). A summary of ofMRI papers, with reference in brackets [10,11,15,17,19,20,34,47,48,49,50,51,52,53,54,55,56,57,58,59,60,61,62,63,64], related to sensory processing.

We briefly describe sensory processing and how the brain elicits appropriate behavioral, emotional, and physiological responses through sensory information from one’s own body and environment (Figure 3A). When sensory signals are sensed in the periphery and transmitted to the brain, sensory perception is easily affected by brain states, such as anesthesia, sleep, wakefulness, and arousal [35,36,37,38]. To have an accurate understanding of the dynamic environment, the brain needs to interpret bottom-up sensory signal based on locomotion, prior experiences, etc., which we can call top-down modulation [39]. For example, animals usually perceive external sensory stimuli along with voluntary movements [40,41], and the sensory processing during voluntary movement actively integrates motor signal with sensory information to cancel self-generated sensory input and to facilitate the extraction of new information from sensory input [40,41]. For survival, it is critical to evaluate the current environment, to predict outcomes, and to elicit adaptive responses based on prior experiences [42,43]. Learning the associations among stimuli, responses, and outcomes results in behavioral patterns to stimuli, such as Pavlovian, goal-directed, and habitual responses [44,45,46]. Pavlovian and classical conditioning induces involuntary and inflexible responses, such as freezing or salivating to a neutral stimulus. In contrast, instrumental conditioning increases or decreases voluntary and flexible behaviors such as lever-pressing for reward-related stimuli. Optogenetic fMRI is commonly used for investigating sensorimotor integration and increasingly for neural circuits subserving cognitive functions. A summary of ofMRI papers related to sensory processing is reported in Figure 3B.

### 4.1. Somatosensory Circuits

#### 4.1.1. Cortical Output of S1 Pyramidal Neurons

The role of S1 in sensory processing is known to receive peripheral somatosensory inputs for sensory discrimination and is also known to integrate sensory and motor signals for skillful movement [65,66]. In rodent fMRI studies, peripheral electrical stimulation showed bottom-up somatosensory processing in thalamo-cortical (TC), cortico-thalamic (CT), and cortico-cortical (CC) circuits: VP→S1→PO/M1 [15]. By utilizing a 250 ms temporal resolution, somatosensory fMRI can differentiate the laminar response in S1, in which BOLD activity appears at layer 4, spreads to layers 2/3 and 5, and finally projects to other regions via CT and CC circuit [15].

Optogenetic activation of S1 through ChR2 expression under various promotors, such as Thy1, CaMKII, or EF1a, induced positive fMRI responses, calcium influx, and electrical signals in the stimulated S1 region [11,17,47,48,49,50]. Additionally, positive fMRI responses were detected at the sites of long-range projection from S1 including the secondary somatosensory cortex (S2), M1, striatum, thalamus, and contralateral S1 [11,17,47]. Similarly, optogenetic silencing of S1 induced negative fMRI responses at S1, S2, M1, striatum, VP, and PO [19]. These data indicate that fMRI combined with optogenetics is suitable for studying brain-wide neural networks including CT and CC circuits.

In particular, somatosensory-evoked fMRI with optogenetic silencing provides a powerful approach to dissecting somatosensory circuits [19]. Optogenetic silencing in S1 suppressed somatosensory-evoked fMRI responses in the downstream circuits (S1→PO/M1) but maintained responses in the upstream regions (VP→S1). This suggests that the VP is involved in the transmission of sensory information to S1, whereas the PO receives sensory information from S1 and integrates dynamic spatiotemporal signals [67]. In addition, peripheral stimulation induced fMRI responses in layer 2/3 of M1 and the modulatory effects of S1 silencing also occurred in layer 2/3 of M1, suggesting that layer 2/3 of M1 receives CC input from S1 [15,19].

S1 of both hemispheres are connected via the corpus callosum. Optogenetic activation of axonal projections from S1 in the corpus callosum induced antidromic and orthodromic fMRI and calcium responses in both hemispheres [50]. The antidromic response of S1 was stronger than the orthodromic response and spread to the ipsilateral motor cortex and thalamus. These data indicate that the antidromic response is not negligible in fMRI studies with high irradiation power. In addition, the orthodromic response was dependent on the frequency of the optogenetic stimulation: low-frequency stimulations induced excitatory effects, while high-frequency stimulations induced excitatory followed by inhibitory effects. These data provide new perspectives in interpreting the function of the corpus callosum in the interhemispheric excitatory-inhibitory balance.

#### 4.1.2. Brain States and Thalamic Modulation

The thalamus and cortex are interconnected by reciprocal projections, and these interactions are well-known to be critical for brain states affecting sensory perception, such as anesthesia, sleep, wakefulness, arousal, etc. [35,36,37,38]. Interestingly, both thalamus and cortex have distinct activity patterns depending on brain state [35,36]. Thalamic firing shows two distinct patterns: burst firing during sleep and tonic firing during wakefulness. In contrast, the cortex shows slow-wave oscillations with large amplitude during quiet wakefulness or sleep states, but fast oscillation with lower amplitude during dynamic behaviors with active sensory sensing. ofMRI thus allows for the investigation of the spatiotemporal coding of brain-wide thalamocortical propagation and processing through frequency-dependent stimulation of the thalamus. 

***Ventral posterior nucleus:*** It is well known that optogenetic activation of VP, a first-order somatosensory thalamic relay, can mimic brain states [35]. As the frequency of optogenetic stimulation increased, the spiking patterns of VP changed from thalamic bursting (sleep/anesthesia state) to tonic activity (awake/arousal state) [56]. Furthermore, optogenetic stimulation of CaMKII neurons showed a frequency-dependent fMRI response [56,57,58]. Low-frequency (1 Hz or long inter-spike intervals of 125ms) stimulation induced bilateral fMRI responses at S1 and related cortical regions connected through polysynaptic propagation. In contrast, high-frequency (5–40 Hz or short inter-spike intervals of 50ms) stimulation induced fMRI response only on the ipsilateral side of S1 and S2. Additionally, in both fMRI and electrophysiological recording, low-frequency stimulation of VP increased interhemispheric functional connectivity in S1 [57]. These results suggest that the thalamus, under low-frequency modulation, elicits brain-wide activity and can potentially explain the neural mechanism of resting-state fMRI.

***Central thalamus and submedial thalamic nucleus:*** Central thalamus is also involved in consciousness such as arousal, attention, awareness, and goal-directed behavior [68]. The function of the central thalamus on cortical excitation–inhibition balance and brain state was investigated with EEG, electrophysiological recording, and ofMRI [59]. In forebrain EEG, low-frequency stimulation of central thalamus CaMKII neurons induced slow-wave oscillations related to the loss of consciousness and behavioral arrest, whereas high-frequency stimulations induced fast oscillations related to awake state and behavioral arousal. Optogenetic activation resulted in both positive and negative fMRI responses in the cortex depending on stimulation frequency. Low-frequency (10 Hz) stimulations of the central thalamus suppressed the basal neural activity of S1 and induced negative fMRI responses, while high-frequency (40 and 100 Hz) stimulations increased neural activity in S1 and induced positive fMRI responses.

Interestingly, optogenetic modulation of the axonal projection terminal from the submedial thalamic nucleus to the orbitofrontal cortex (OFC) showed different polarities in cortical fMRI responses depending on stimulation frequency [60]. Low-frequency (10 Hz) stimulation induced negative fMRI responses in the overall cortical regions except for the stimulated site of OFC, while high-frequency (40 Hz) stimulation induced positive fMRI responses only in ipsilateral cortical regions. However, soma activation of OFC and thalamus failed to reproduce the frequency-dependent fMRI pattern induced by axon terminal activation. These cortical inhibitions by low-frequency modulation of thalamic terminals indicate the involvement of inhibitory mechanism via cortical interneurons, thalamic reticular nucleus, or other GABAergic population (e.g., zona incerta) [59,60]. 

#### 4.1.3. Top-Down Modulation of Sensory Processing

Top-down modulation prioritizes sensory information, adjusts relevant neural pathways, and determines behavior responses, which are associated with M1 and medial prefrontal cortex (mPFC).

***Primary motor cortex:*** In human studies, M1 stimulation is generally recommended as a treatment for patients with motor disorders and sensorimotor dysfunction such as Parkinson’s disease, chronic refractory pain, post-stroke recovery, and tinnitus [69], suggesting that M1 is closely involved throughout the sensorimotor system. ofMRI studies detected brain-wide circuitry driven by M1 stimulation using Thy1-ChR2 mice and AAV-CaMKII-ChR2 [10,15,51,52]. Optogenetic activation of M1 in Thy1-ChR2 mice showed sequential neural information flow from M1 to the somatosensory pathway (M1→S1→S2/VP) [15]. Moreover, optogenetic silencing of M1 induced negative fMRI responses in the somatosensory network, particularly in layer 2/3 and layer 5 of S1 [19]. These data indicate that the somatosensory system directly receives information from M1. 

The optogenetic activation of M1 was also used to measure altered brain circuity of M1 after stroke induction by transient middle cerebral artery occlusion (MCAO), resulting in infarction in S1 regions [52]. In the acute phase (3 days after MCAO), optogenetic activation of M1 failed to increase the fMRI responses even at the stimulated M1 site but rather induced negative fMRI responses. However, at 15 days post-MCAO, M1 stimulation re-induced positive fMRI responses at the M1 site with reduced activation in S1 and thalamus (PO, VP, and ventral anterior-lateral thalamus (VAL)), compared to pre-stroke. Repeated optogenetic stimulation of M1 restored the activation of S1 and thalamus, which positively correlated with improved sensorimotor coordination. These findings provide brain-wide evidence that M1 is involved in top-down modulation in sensory processing. In addition, ofMRI in stroke models allowed for in vivo investigation of which downstream M1 circuits were involved in stroke recovery following M1 stimulation treatment. 

***Medial prefrontal cortex:*** The mPFC plays an important role in assessing the current environment, predicting outcomes, and deriving adaptive responses based on prior experiences [42,43]. For example, the mPFC is involved in discriminating sensory cues related to reward/punishment, predicting safety, and inducing reward-seeking or fear responses [70]. In other words, the function of the mPFC is more related to discriminating positive and negative valences of sensory cues (affective-motivational aspect) rather than discriminating sensory modality (sensory-discriminative aspect). In rodent studies, the prelimbic cortex (PrL), infralimbic cortex (IL), and ACC compose the mPFC. Unlike the top-down processing of the M1 that induces strong modulation of the somatosensory network (S1, VP, and PO), optogenetic activation of the ACC and IL induces large fMRI responses in motivation-related brain regions, including dorsal and ventral striatum rather than the somatosensory network [20,53,54]. 

In pain research, the ACC is well-known to be involved in the affective-motivational aspect of pain, while the S1 is important for the sensory-discriminative aspect of pain, such as spatiotemporal information about intensity and quality [71]. In the chronic inflammatory pain model, increased spontaneous ACC activity and enhanced interregional connectivity were found when silencing the ACC with ofMRI (Figure 2B) [20]. Interestingly, the response to ACC silencing was stronger within brain regions involved in threat coping, including striatum, periaqueductal gray, and superior colliculus rather than the somatosensory regions (Figure 2C). Given that the ACC is known to be involved in fear and defensive responses to threat signals caused by visual, odor, or pain, the function of the ACC is essential for motivation to react to sensory stimuli rather than sensory transmission.

In addition, the mPFC has a bidirectional effect on the dopamine-related motivation levels depending on the frequency of stimulation [34,54,55]. Optogenetic self-stimulation of mPFC (AAV-hSyn-ChR2 into IL; 25 Hz) serves as a reward in rodents and induces positively reinforced behaviors for receiving optogenetic stimulation [34,54]. Activation of the IL (25Hz) increased fMRI responses in multiple motivation-related regions, including the anterior insular area, anterior thalamus, ventral striatum, and hypothalamus, which were positively correlated with the level of reinforced behaviors [34,54]. On the other hand, continuous activation of the IL with step-function opsin suppressed both reward-related behavior and the striatal fMRI response to stimulation of midbrain dopaminergic neuron [55]. These results also suggest the importance of the mPFC in the affective-motivational dimension.

#### 4.1.4. Response Output to Sensory Stimuli

Conditioning refers to the acquisition of a new response to stimuli. It can be divided into classical and instrumental conditioning [44,45,46]. The amygdala and striatum are known to be related to classical and instrumental conditioning, respectively [45,46,72]. Since both amygdala and striatum are composed of functionally heterogeneous neurons and multiple subregions with complex neural circuitry, ofMRI has an advantage in targeting molecular and genetic cell types in a specific region to investigate whole-brain circuity [73,74,75,76,77].

***Central amygdala:*** The central nucleus of the amygdala (CeA) receives direct nociceptive input via the parabrachial nucleus and integrates it with information about context and cue and internal state to generate fear memory, negative emotional responses, and Pavlovian conditioning [74,78]. Two non-overlapping subpopulations of GABAergic neurons in CeA are known to have opposing functions in processing aversive stimuli and associative fear learning: protein kinase C delta-expressing neuron (CeA-PKCδ) and somatostatin-expressing neuron (CeA-SOM) [74,75,79]. 

ofMRI combined with nociceptive heat stimuli demonstrated differential roles of CeA-PKCδ and CeA-SOM neurons on bottom-up or top-down interactions at the network level [61]. Optogenetic activation of CeA-PKCδ neurons reduced heat-evoked fMRI responses in the brainstem, thalamus, and limbic regions including amygdala, hypothalamus, and basal forebrain, while slightly increasing S1 responses. On the other hand, CeA-SOM activation enhanced heat-evoked fMRI responses in the limbic regions and S1. During heat stimulation, the optogenetic activation of CeA-PKCδ neurons reduced cortical functional connectivity with the basal forebrain and hypothalamus, while activating CeA-SOM reduced functional connectivity of S1 (with thalamus, cingulate, hippocampus, and amygdala) and brain stem (with hippocampus and M1). 

***Striatum:*** The striatum plays an important role in limbic functions related to behavioral and emotional responses, particularly in instrumental behaviors such as goal-directed action and stimuli-driven habits [45,80]. The dorsal striatum (dorsal part of caudate putamen), the main input nucleus of the basal ganglia, receives inputs from the cerebral cortex and is known to project exclusively within the basal ganglia network [73,80]. The GABAergic medium spiny neurons are the major striatal output and classified as direct (striatonigral) or indirect (striatopallidal) antagonistic pathways. The striatal direct pathway expressing D1 dopamine receptors projects to and inhibits the inhibitory substantia nigra pars reticulate (SNr) and prompt motor initiation, whereas the striatal indirect pathway expressing D2 dopamine receptors projects to and inhibits the globus pallidus. The striatal indirect pathway also disinhibits the subthalamic nucleus (STN), which in turn activates SNr and leads to movement termination. Furthermore, the striatum can be divided into two subregions: dorsolateral striatum (DLS) and dorsomedial striatum (DMS). The DLS receives inputs mainly from the S1 and is related to stimuli-driven habitual responses, while the DMS receives inputs mainly from the mPFC and is related to the goal-directed actions [73,76,77]. Although their basal ganglia connections are well-known, how they affect or are affected by other brain networks is unclear. 

Lee et al. (2016, 2017) utilized ofMRI by stimulating neurons specific to either the direct or indirect pathways with D1-Cre and D2-Cre mice, respectively [62,63]. The direct pathway (D1) stimulation induced positive fMRI responses in the thalamus and motor cortex, whereas the indirect pathway (D2) stimulation resulted in negative responses in the same areas, which suggested the direct pathway’s role in movement initiation and indirect pathway’s role in movement termination. Interestingly, D1 stimulation also resulted in positive fMRI responses in the globus pallidus internal and SNr, which should be inhibited by D1 medium spiny neurons, and in STN and globus pallidus external, which are part of the indirect pathway. In contrast, D2 stimulation resulted in the negative fMRI response in STN, which should be classically disinhibited. Neural recording in the observed areas reflected the fMRI response. These results show ofMRI capability to map modulation from other pathways or feedback mechanisms, particularly when using sustained stimulation durations (20 s). The positive fMRI and increased firing of STN and globus pallidus internal neurons from D1 stimulation were proposed to be due to the feedback hyperdirect pathway. As D1 stimulation increased motor cortex activity, increased excitatory projections from the motor cortex to STN may explain the positive fMRI from D1 stimulation. On the other hand, D2 stimulation reduced motor cortex activity, which reduced the activation of STN. In an ofMRI study of D1/D2 expressing neurons in the ventral striatum (ventral part of caudate putamen), positive fMRI response was observed in the same basal ganglia thalamocortical network, along with the anterior cingulate cortex, infralimbic area, dentate gyrus, and cerebellum [64]. Although these findings are speculative, we see the advantage of using ofMRI in investigating the complex basal ganglia network and its interactions with different networks, which can promote further investigations.

### 4.2. Other Sensory Modalities

Optogenetic activation of the visual cortex (V1) elicited BOLD fMRI activity in the main visual pathways such as V1, secondary visual cortex (V2), superior colliculus (SC), and auditory cortex [81]. Optogenetic V1 stimulation did not induce inferior colliculus (IC) response but enhanced IC activity during simultaneous auditory and V1 stimulation, showing evidence for cross-modal sensory interplay [81]. Optogenetic fMRI was also used to investigate how the midbrain vestibular nucleus (MVN), an area involved in coordination of balance and movement, affects both visual and auditory processing [82]. Anatomical tracing studies have shown that the MVN receives inputs from all sensory modalities suggesting an important role in multisensory integration. Stimulation of the MVN alone showed widespread fMRI response related to auditory, somatomotor, and visual circuits along with areas of cognition such as hippocampus, retrosplenial cortex, and ACC. When combined with simultaneous auditory stimulation, MVN stimulation enhanced activity within the ipsilateral IC, medial geniculate body, and auditory cortex. Similarly, simultaneous binocular visual stimulation showed enhanced ipsilateral lateral posterior thalamus (LP), lateral geniculate nucleus (LGN), and visual cortex. Interestingly, the SC contralateral to the stimulated MVN showed enhanced response compared to the ipsilateral SC, showing a differential hemispheric effect on sensory processing by the MVN. 

Several other ofMRI studies with activation in sensory areas have been conducted and provide potential future sensory-modulation studies. Stimulation of the hippocampus, a region particularly studied in spatial navigation and memory in rodents, has shown positive BOLD fMRI response within the S1 and caudate putamen [83]. Although no direct monosynaptic hippocampal–somatosensory connection exists, coupling between the S1 and hippocampus was observed during slow wave sleep [84], suggesting that ofMRI is capable of detecting polysynaptic connections of the hippocampal network [85]. Other studies have shown visual cortex activation following hippocampus stimulation in a region- (intermediate vs. dorsal) or frequency- (low vs. high) dependent manner [86,87]. Visual cues play a pivotal role in hippocampal activity during spatial navigation, and thus, fMRI activity in the visual cortex is expected [88,89,90]. These studies open up further visual or somatosensory-modulated effect studies of the hippocampal network.

Other studies include the cerebellum and dorsal raphe nucleus ofMRI [91,92]. Cerebellar stimulation was also found to elicit BOLD fMRI activity in the somatosensory and motor cortex among various other forebrain and midbrain regions [91]. The cerebellum receives inputs carrying sensory, proprioceptive, and higher-order information, but its downstream forebrain efferent connections and their modulation on sensorimotor integration is not well-understood. The dorsal raphe nucleus (DRN) is the main serotonin output of the brain, which is involved in mood, memory, circadian rhythm, and feeding and is predominantly studied for its role in depression and stress. Interestingly, optogenetic activation of the DRN elicited positive response in the stimulated site but negative response within the whole brain including the somatosensory and visual cortex [92]. Taken together, these studies show the ability of ofMRI to map sensory cortex activity by stimulating non-sensory or higher-order areas.

## 5. Caution for ofMRI

Although the use of optogenetic fMRI allows for brain-wide network investigation, several cautions must be taken in interpreting its results.

### 5.1. Relationship between Neural Activity and fMRI Response

ofMRI reports the BOLD signal, which is an indirect measure of neural activity. BOLD fMRI signal relies on changes in the deoxyhemoglobin concentration in an area and thus is a complicated interplay between functional hyperemia, the cerebral metabolic rate of oxygen (CMRO_2_), and blood volume [93]. In addition, optogenetically activating astrocytes induced a BOLD response without neural activation, which was found to be due to increased CMRO_2_ [94]. Increased CBF, which suggests positive BOLD, was reported when cortical stimulation of VGAT-ChR2 transgenic mice was briefly stimulated [95]. When stimulation duration was sufficiently long for reaching a steady-state condition, negative BOLD response to optogenetic stimulation of VGAT-ChR2 was dominant in cortical and downstream thalamic regions [17]. Thus, the BOLD signal is a net effect of excitation and inhibition balance within the observed area, as measured by local field potential [96,97]. Fortunately, most ofMRI studies supplement their findings with either neural recordings or calcium imaging of the stimulated and/or observed projection sites and have reported positive BOLD with increased neural activity and vice versa. 

### 5.2. Sensitivity Issue

Rodent fMRI particularly suffers from low sensitivity due to the nature of the BOLD fMRI signal. A single neural response is not sufficient to see an fMRI signal, and thus, small areas and/or sparse neural populations may not elicit sufficient neurovascular coupling for fMRI detection. ofMRI failed to significantly report brain-wide response when a promoter targeting only dopaminergic neurons within the small ventral tegmental area was used [98]. On the other hand, using a promoter that targets all pyramidal neurons in the same area resulted in a significant brain-wide response [98]. These factors may limit ofMRI response restricted to the strongest connected areas and may underreport brain-wide response to a stimulated area. Increasing the signal-to-noise ratio by using a stronger magnet (≥7 Tesla) or by conducting awake ofMRI has shown brain-wide fMRI response in functionally connected areas [55,99]. Furthermore, the small rodent brain (415 mm^3^ in mice [100] and 1765 mm^3^ in rats [101]) requires a very high spatial resolution, which can reduce sensitivity. Rodent fMRI is mostly non-isotropic with a spatial resolution of 0.15–0.5 mm and 0.4–1.0 mm slice thickness and is conducted with a temporal resolution between 1–2 s. As a result, the majority of ofMRI studies have been conducted at a high field of 9.4 Tesla, which compensates for the reduced sensitivity from high spatial resolution. Higher spatial resolution and faster temporal resolution with high sensitivity is achievable when using an ultra-high field 15.2 Tesla magnet [15]. Although optogenetics allows for specific neural population targeting, it can be offset by the BOLD fMRI low sensitivity nature, and thus, information on a target population, magnet strength, and anesthesia must be taken into account. In addition, targeting of a small neural population may require a higher laser power for sufficient activation to elicit activity in connected long-range regions [91]. However, this may lead to tissue heating and heating artifacts, which confounds ofMRI interpretation.

### 5.3. Heating Issue

Optogenetics uses light to photoactivate an area of neurons. Depending on the irradiance of the light, heating can induce a positive BOLD response without neural activation by causing changes in the arteriolar smooth muscle [102], or a positive BOLD response accompanied by nearby negative BOLD voxels [103,104,105]. A negative fMRI signal was also reported at the fiber site due to heating-induced frequency shifting within the hippocampus [105]. It is thus imperative to know whether the heat-induced fMRI signals exist within the brain when interpreting ofMRI results. A common control is to check whether the light irradiance used can induce heating artifacts on naïve mice that do not express the opsin. In addition, comparing the BOLD signal between the stimulated site and projection site may help detect heating artifacts, and sensory/optogenetic stimulation was found to elicit different signal shapes [106]. 

Heating artifacts and false BOLD response can also be caused by tonic stimulation. In the prefrontal cortex, temperature and neural firing rate increase were detected after 30 s of continuous 60 mW/mm^2^ (5 mW with 200 µm fiber) intensity light illumination [107]. In opsin-free mice, a modest suppression of medial spinal neuron activity in the striatum was seen from 3 mW continuous illumination of 5 s [108]. Similarly, behavior changes were seen when higher intensity light was used, but behavioral effect ceased when a high intensity was given at 20 Hz. All ofMRI experiments reviewed have used laser power within 3–10 mW with varying irradiances due to differing optic fiber diameters and were conducted with pulsed stimulations at varying frequencies unlike the continuous illumination protocol mentioned above. Thus, optogenetic stimulation parameters must be considered when interpreting ofMRI results as well. 

## 6. Conclusions and Future Prospects

ofMRI to date is the only in vivo method to causally investigate functional connectivity within the whole brain. It has been proven to be a valuable tool in investigating the brain dynamics and connectivity of genetically and topologically defined neural populations. Since its first seminal ofMRI study [10], there has been a rapid growth in ofMRI research. The majority of early ofMRI studies have been limited to the functional mapping of a stimulated region, but improvements in experimental design and acquisition methods have expanded ofMRI applicability in mapping spatiotemporal propagation and processing along with how disease conditions affect brain circuitry as seen in seizures [109,110], Alzheimer’s Disease [111], stroke [52], and chronic pain [20]. Future studies can further delineate the frequency-dependent brain responses of deep-brain nuclei and further characterize circuit changes in other diseases. Advances and improvements in awake behaving ofMRI studies can potentially expand our understanding of how the brain dynamically responds to a task. In particular, the use of silencing ofMRI has the potential to better understand how a brain region is integrated within a network. Silencing ofMRI in disease models and/or awake studies may elucidate the functional importance and plasticity of a silenced region within the brain network. While there are still caution and limitations to ofMRI, it is a valuable method that expands the neuroscience toolbox and helps us to gain further comprehension of the brain network.

## Data Availability

Not applicable.
